# Raman Study of Central Metals and Substituents Effects on Metal Phthalocyanines

**DOI:** 10.3390/nano16160982

**Published:** 2026-08-10

**Authors:** Xiaofang Zhang, Dongliang Tian, Rongming Wang

**Affiliations:** 1 Beijing Key Laboratory for Magneto-Photoelectrical Composite and Interface Science, The State Key Laboratory for Advanced Metals and Materials, School of Mathematics and Physics, University of Science and Technology Beijing, Beijing 100083, China; xfzhang926@ustb.edu.cn; 2 Key Laboratory of Bio-Inspired Smart Interfacial Science and Technology, School of Chemistry, Beihang University, Beijing 100191, China; tiandl@buaa.edu.cn

**Keywords:** Raman, metal phthalocyanine, central metal, substituents effects

## Abstract

Metal phthalocyanines (MPcs) possess outstanding thermal stability and versatile optical, catalytic, and semiconducting properties. Here, the effects of central metal ions, peripheral substituents, and substitution positions on the vibrational properties of MPcs were systematically investigated using Raman spectroscopy. The characteristic Raman band near 1530 cm^−1^ exhibits systematic shifts with changing metal centers (NiPc > CoPc > CuPc > ZnPc), reflecting differences in metal-ligand interactions and electronic structure. Peripheral substituents and their positions further influence the Raman response through electronic effects, vibrational coupling, and resonance enhancement. In particular, four-substituted MPcs show greater Raman shifts than their three-substituted counterparts. The results reveal clear correlations between molecular structure and Raman characteristics, providing a useful framework for understanding and designing functional phthalocyanine-based materials.

## 1. Introduction

Phthalocyanines (Pcs) are aromatic macrocyclic compounds containing an 18 π-electron conjugated system along the inner macrocyclic perimeter, which endows them with unique electronic and optical properties [1,2,3,4]. The central N_4_ cavity of the phthalocyanine macrocycle has a diameter of approximately 2.7 Å [1,2], which enables coordination with a wide range of metal ions [5,6,7,8,9,10,11]. Besides conventional monomeric MPcs, the central N_4_ cavity can coordinate suitable metal atoms to form a variety of higher-order architectures, including double-decker (bis-phthalocyaninato), triple-decker and multiple-decker sandwich complexes, particularly for lanthanide atoms [1,12,13,14,15,16,17]. MPcs are representative functional molecular materials that have attracted extensive interest in molecular electronics, chemical sensing, catalysis, self-assembled thin films, and interfacial functional systems [18,19,20,21,22,23]. A detailed understanding of their vibrational characteristics and metal-ligand interactions are therefore essential for the structural characterization and rational design of phthalocyanine-based micro/nanostructured and interfacial functional materials.

The properties of Pcs can be tailored through variation in the central metal ion, axial coordination, peripheral substitution, modification of the macrocyclic framework, and construction of higher-order architectures such as bis (Pcs) and polymeric Pcs. Among these, the identity of the central metal ion and the nature as well as the substitution pattern of peripheral groups are two key factors to precisely tuned the physicochemical properties of MPcs. For example, modulation of the central metal and substituents influences the electronic structure of the M–N_4_ active center, which is directly related to catalytic activity and selectivity, substituent-induced electronic effects can alter molecular conjugation, charge transport, and intermolecular packing [5,24,25,26,27]. Transition metals such as Fe, Co, and Ni coordinate within the Pc macrocycle through a combination of covalent and coordination interactions, forming thermodynamically stable complexes with distinct electronic configurations. The theoretical and experimental investigation of molecular, electronic structures and vibrational spectrum for FePc, CoPc, NiPc, CuP, and so on, have been performed [28,29,30,31]. In addition, substitution of peripheral hydrogen atoms with functional groups-such as amino, nitro, or bulky aryl moieties-provides a versatile strategy for modulating the molecular structure and properties of MPcs [32,33,34]. These substituents can markedly influence solubility, electronic structure, and intermolecular interactions, thereby enabling fine control over material performance [35,36,37,38,39]. Such structural tunability offers broad opportunities for designing MPc-based materials tailored for advanced applications, including chemical sensing, organic electronics, photodynamic therapy, and artificial photosynthesis. Despite these advances, the rational design of MPc-based systems with targeted functionalities still requires a comprehensive understanding of how central metal ions and peripheral substituents collectively govern molecular structure, bonding characteristics, and electronic behavior.

Raman spectroscopy, owing to its non-destructive nature and high sensitivity to molecular vibrations, has emerged as a powerful analytical tool for probing structure-property relationships. It provides detailed insights into molecular vibrational modes, which are intrinsically associated with bonding configurations, molecular symmetry, and the electronic environment [37,38,39,40,41]. A key advantage of Raman spectroscopy lies in its sensitivity to changes in molecular polarizability during vibrational processes, making it particularly effective for investigating the fundamental characteristics of MPcs. For example, it can elucidate the influence of central metal ions on the electron density distribution within the Pc macrocycle, as reflected by measurable shifts in vibrational frequencies. Likewise, it reveals the effects of electron-donating and electron-withdrawing substituents on the conjugated π-electron system, leading to variations in Raman band positions and intensities. Furthermore, Raman spectroscopy can capture the positional dependence of substituents on molecular symmetry and vibrational coupling, often manifested as band splitting or changes in relative intensity. 

In this study, we conduct a systematic investigation of the Raman spectral characteristics of MPc compounds featuring four distinct central metals (Ni, Co, Cu, and Zn), four peripheral substituents (amino, nitro, p-tert-butylphenoxy, and 5-isopropyl-2-methylphenoxy), and two substitution patterns (three and four positions). By correlating variations in Raman peak positions and intensities with changes in molecular polarizability, electronic effects, and symmetry, we aim to elucidate the fundamental mechanisms governing Raman shifts in these derivatives. The insights gained from this work are expected to provide a solid theoretical basis for the rational molecular design, functional optimization, and practical application of next-generation phthalocyanine-based materials.

## 2. Materials and Methods

### 2.1. Synthesis of MPcs via a Template-Based Method

In this study, a series of three and four substituted MPcs, actually complexed compounds of the different isomers, incorporating central metals (M = Ni, Co, Cu, and Zn) were synthesized, purified through column chromatography (just for achieving the complexed compounds with different isomers) and fully characterized in our previous studies [42,43,44]. The selected substituents-amino (–NH_2_), nitro (–NO_2_), p-tert-butylphenoxy (C_10_H_13_O–), and 5-isopropyl-2-methylphenoxy (C_10_H_13_O–) were deliberately chosen to represent distinct electronic characteristics. These groups were introduced at either the 3 or 4 positions of the Pc macrocycle, yielding a structurally diverse set of derivatives. A systematic nomenclature was adopted to distinguish the compounds. For the 3-substituted series, representatives include MPc-3a, MPc-3n, MPc-3p, and MPc-3i (where a, n, p, and i denote amino, nitro, p-tert-butylphenoxy, and 5-isopropyl-2-methylphenoxy substituents, respectively), with M = Ni, Co, Cu, and Zn. An analogous naming scheme was applied to the corresponding 4-substituted counterparts (MPc-4a, MPc-4n, MPc-4p, and MPc-4i). Following synthesis and extraction, all MPc compounds were obtained as fine powders suitable for subsequent characterization. Raman spectroscopy was then employed as a robust analytical technique to systematically investigate the vibrational properties of the synthesized materials.

### 2.2. Spectral Measurements

Raman spectra were acquired using a Horiba LabRAM HR800 Raman spectrometer (HORIBA Jobin Yvon SAS, Villeneuve d’Ascq, France) equipped with a 532 nm semiconductor laser with 100 mW as the excitation source. The instrument provided a spectral resolution better than 2 cm^−1^. A Leica 50× long working distance (LWD) objective was employed for MPc samples. The spectral range was set from 300 to 1800 cm^−1^, covering the characteristic vibrational modes associated with functional groups and molecular frameworks in MPc compounds. For each measurement, exposure lasts for 10 s and is repeated 3 times. The final spectrum was obtained by averaging the three independent measurements to improve the signal-to-noise ratio and ensure spectral reproducibility. To enable a more direct comparison among Pcs compounds containing different central metal atoms (Ni, Co, Cu, and Zn) and substituents, all spectra were normalized prior to analysis. In addition, UV–Vis absorption spectra were recorded using a Lambda 950 spectrophotometer (PerkinElmer, Waltham, MA, USA) in the wavelength range of 250–800 nm. Measurements were carried out in CHCl_3_ solution for the as-synthesized MPcs derivatives with different central metals and substitution positions. 

## 3. Results and Discussion

### 3.1. Morphology and Structure of MPcs

Pcs are a class of macrocyclic compounds renowned for their exceptional thermal and chemical stability, as well as their outstanding optical, catalytic, and semiconducting properties. However, their practical application is often hindered by poor solubility in both aqueous and organic media, primarily arising from strong π–π stacking interactions between their highly planar conjugated structures. These pronounced intermolecular interactions impede processing, separation, and purification, thereby limiting large-scale utilization. To address this limitation, two principal modification strategies are commonly employed: tuning the central metal and engineering peripheral substituents. The central metal ion, embedded within the Pc macrocycle, and the substituents attached to its periphery play crucial roles in governing the physicochemical properties of the molecule. Notably, even subtle modifications to the parent Pc framework can induce significant changes in its electronic structure and vibrational behavior.

**Figure 1:**
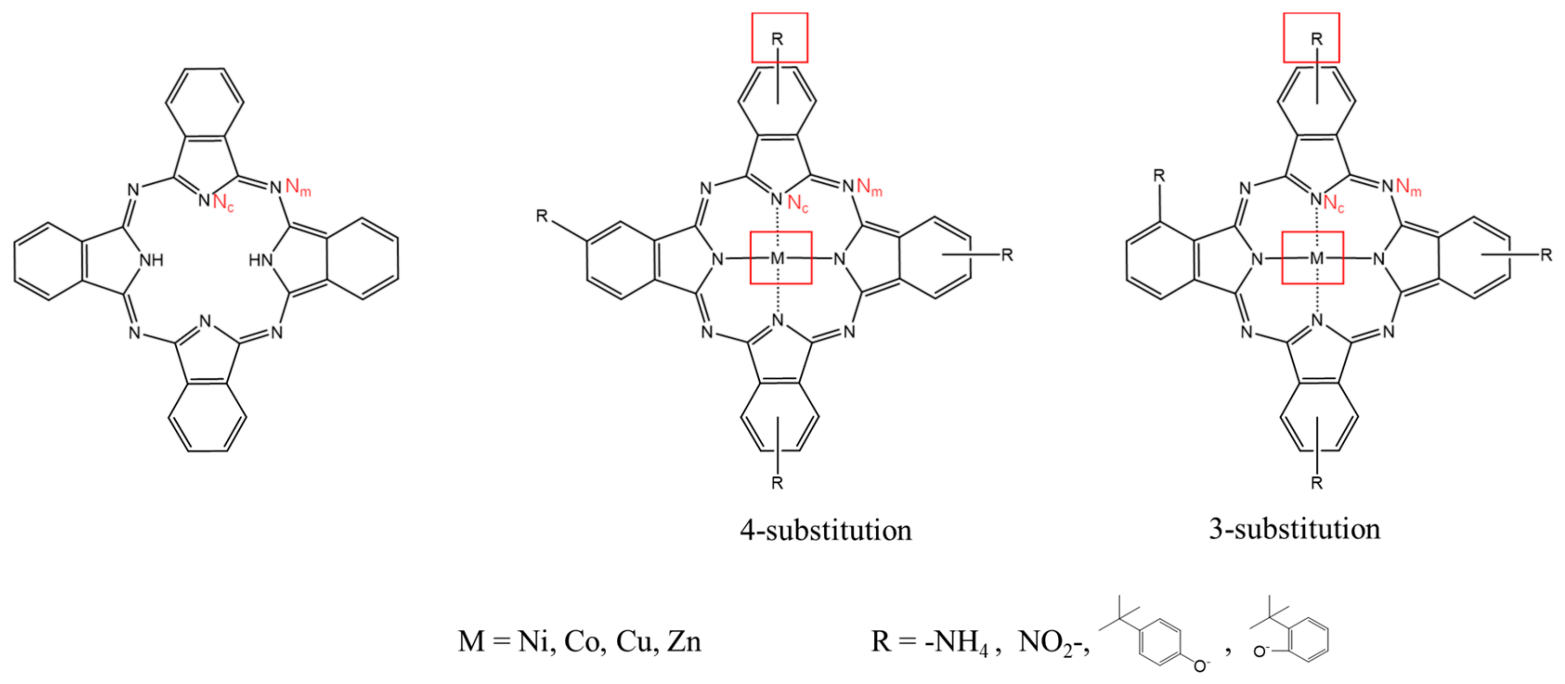
Structural representations of phthalocyanine (Pc) and metal phthalocyanines (MPcs) with varying central metals and peripheral substituents. We defined the positions of the key N atoms, i.e., N_c_ and N_m_, in the figure.

The structural features of Pcs and MPcs, which vary in terms of the central metal atom and peripheral substituents, are illustrated in Figure 1. To enhance solubility, substituents with higher solvent affinity were introduced onto the outer rings of the Pc macrocycle, aiming to disrupt the strong π–π- stacking interactions between planar Pc molecules. The geometric parameters of these MPc-4i compounds, specifically the N–-M (where M represents the central metal), were calculated using Chem3D Ultra 8.0 and summarized in Table 1. An in-depth analysis of the N–M distances in the MPc-4i series reveals a distinct trend: the N–M distance increases in the order N–Ni (1.826 Å) < N–Co (1.836 Å) < N–Cu (1.846 Å) < N–Zn (1.926 Å). A similar order is observed for the N–M distances across the same series of MPcs with central metal atoms Ni, Co, Cu, and Zn without substituent group [29,45,46,47]. This trend can be rationalized in terms of the metal-ligand bond distances, which increase as the electrostatic attraction between the central metal atoms and the surrounding ligands decreases. The geometric variations described above are expected to exert a direct influence on the vibrational behavior of MPc molecules. From a fundamental perspective, Raman-active vibrational frequencies are governed by the classical relation:

(1) $$\begin{array}{c} v\;\propto \sqrt{\frac{k}{\mu }}\;\;\;\; \end{array}$$

where k is the bond force constant and μ is the reduced mass. Consequently, changes in N–M bond length directly affect bond stiffness and thus vibrational frequencies. In the present system, the increase in N–M bond length from Ni to Zn indicates a gradual weakening of metal-ligand interactions. This reduction in bond strength lowers the effective vibrational characteristics, leading to a red shift (lower wavenumber) in vibrational modes involving the Pc framework, particularly the C–N stretching modes. Meanwhile, the increasing atomic mass of the central metal further contributes to this shift by increasing the reduced mass. In addition, variations in metal-ligand interactions alter the electron density distribution across the conjugated macrocycle, thereby modifying molecular polarizability. These combined effects establish a direct structural origin for the observed Raman spectral differences.

### 3.2. Raman Spectra and UV–Vis of MPcs with Different Central Metals

**Figure 2:**
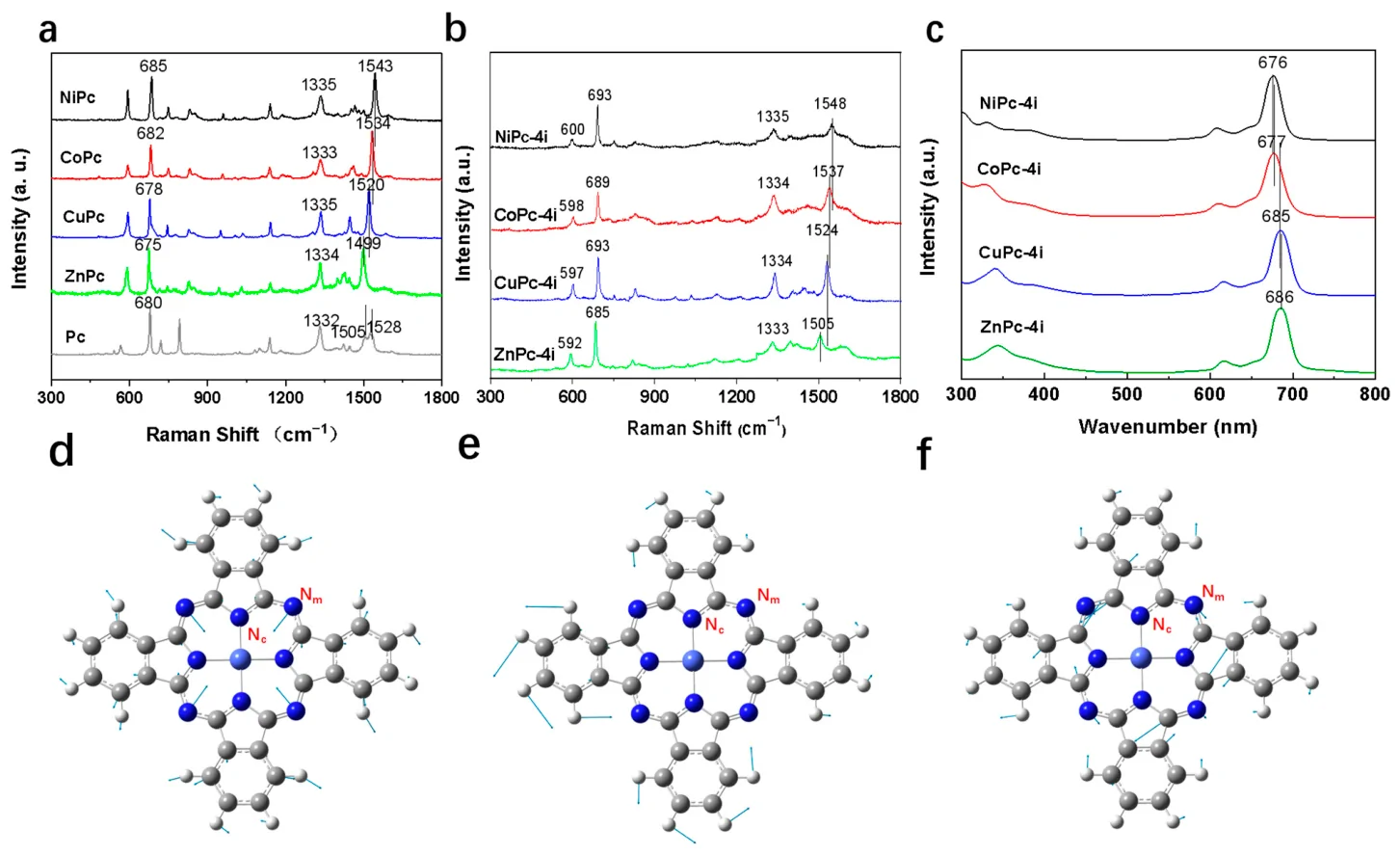
Comparative analysis of Raman spectra for Pcs and MPcs with different central metals and UV–Vis for MPcs with different central metals. (**a**) Raman spectra of Pc, NiPc, CoPc, CuPc, and ZnPc, respectively, highlighting the distinct peaks corresponding to various vibrational modes within the phthalocyanine macrocycle. (**b**) Raman spectra of NiPc-4i, CoPc-4i, CuPc-4i, ZnPc-4i, respectively. (**c**) UV–Vis spectra of NiPc-4i, CoPc-4i, CuPc-4i, and ZnPc-4i, respectively, showcasing the prominent B (Soret) and Q bands indicative of their extended π-conjugated systems. (**d**) The in-plane bending vibrations of C–N_m_–C and C–H of MPcs. (**e**) The in-plane bending vibration of C–H of MPcs. (**f**) The C–N_m_-C stretching vibration and in-plane bending vibration of C–H of MPcs.

Previous research has established that the central metal atom and peripheral substituents are two key factors in modulating the π-conjugated systems of Pc molecules, thereby influencing their electronic distribution and vibrational characteristics. To delve deeper into these effects, the Raman spectra of Pc, MPc (M = Ni, Co, Cu, Zn) (Figure 2a), and MPcs-4i (Figure 2b) and UV–Vis absorption spectra of NiPc-4i, CoPc-4i, CuPc-4i, and ZnPc-4i (Figure 2c) were meticulously collected and analyzed. As shown in Figure 2a, three obvious characteristic Raman bands appear at approximately 690, 1330, and 1530 cm^−1^. To provide theoretical support for the vibrational assignments, DFT calculations were performed on CoPc at the TPSSh level using the ORCA package. The calculated normal modes indicate that the ~690 cm^−1^ band mainly arises from the in-plane bending vibrations of C–N_m_–C and C–H (Figure 2d), the ~1330 cm^−1^ band is dominated by the in-plane bending vibration of C–H (Figure 2e), and the ~1530 cm^−1^ band mainly originates from the C–N_m_–C stretching vibration coupled with the in-plane bending vibration of C–H (Figure 2f). These calculated assignments are consistent with previous literature reports [29,47]. The Raman shifts in the ~690 and ~1330 cm^−1^ bands exhibit only minor variations after metal incorporation, whereas the ~1530 cm^−1^ band displays a much larger shift. This difference indicates that individual vibrational modes respond differently to the central metal ion. Because the ~1530 cm^−1^ band contains a larger contribution from C–N_m_–C stretching vibrations, which are more sensitive to changes in the N–M coordination environment than bending vibrations, its frequency is more strongly influenced by the nature of the central metal. Accordingly, NiPc and CoPc exhibited larger blue shifts than CuPc and ZnPc, consistent with stronger N–M interactions and shorter N–M bond lengths, and increased the vibrational characteristics within the macrocycle. These differences are consistent with variations in metal ionic radius and metal-ligand interactions, which indirectly influence the vibrational behavior of the conjugated aromatic framework [29]. As a result, the Raman shift follows the order: NiPc (1543 cm^−1^) > CoPc (1534 cm^−1^) > CuPc (1520 cm^−1^) > ZnPc (1499 cm^−1^). These results indicate that the vibrational features in this region are sensitive to the presence of the central metal. The order of MPcs with substituent group is NiPc-4i (1548 cm^−1^) > CoPc-4i (1537 cm^−1^) > CuPc-4i (1524 cm^−1^) > ZnPc-4i (1505 cm^−1^) (Figure 2b), the same as NiPc, CoPc, CuPc, ZnPc with p-tert-butylphenoxy-substituted [44]. This trend can be rationalized by considering both bond strength and electronic effects: (1) Metal-ligand bond strength: Ni^2+^ possesses a relatively small ionic radius and high charge density, leading to stronger metal-nitrogen interactions. This enhances the rigidity of the macrocycle and increases the vibrational characteristics of C–N bonds, resulting in higher vibrational frequencies (blue shift). (2) Electron back-donation and d orbital participation: Transition metals such as Ni, Co, and Cu can interact with the π-system of the Pc ring through d–π interactions. Stronger coupling enhances electron delocalization, increasing bond stiffness and Raman frequency. In contrast, Zn^2+^ (d^10^ configuration) lacks effective π-backbonding, resulting in weaker electronic coupling and lower vibrational frequencies. (3) Polarizability effects: Raman intensity is directly related to changes in molecular polarizability. Ni-centered systems exhibit greater polarizability modulation during vibration, leading to stronger and sharper Raman peaks. In contrast, vibrational mode near ~1330 cm^−1^, and the ~690 cm^−1^ band exhibits comparatively minor variations reflecting the influence of the central metal ions. This indicates that low-frequency skeletal deformations are less sensitive to electronic perturbations and are primarily governed by the overall geometric framework rather than localized electronic effects. 

The differences in Raman shifts among these MPcs can be traced back to the variations in the ionic radii, electronic configurations, and polarizabilities of the central metal ions. The electrostatic field strength around the metal center, which is determined by both the ionic charge (Z) and ionic radius (r), plays a crucial role in governing the degree of attraction between the metal and ligand atoms. Metals with a higher charge density, such as Ni^2+^, exert stronger coulombic forces, resulting in shorter and stronger metal-ligand bonds. These trends provide valuable insights into the complex interplay between the central metal and the phthalocyanine ligand, which is essential for the rational design and optimization of functional materials based on MPcs.

In addition to conducting vibrational analysis, UV–Vis absorption spectroscopy of MPcs were also thoroughly examined, as depicted in Figure 2c. All four types of MPcs—namely NiPc-4i, CoPc-4i, CuPc-4i, and ZnPc-4i—display two prominent absorption bands, the B band (also known as the Soret band) around 340 nm and the Q band within the 600–700 nm range. These absorption features are characteristic of the extended π-conjugated system inherent in Pc structures, as documented in references [48,49]. The positions and intensities of these bands are highly sensitive to both the molecular architecture and the electronic environment surrounding the Pc core. The Q band, typically observed near 700 nm, arises from a π–π* electronic transition between the a_1u_ (highest occupied molecular orbital, HOMO) and e_g_ (lowest unoccupied molecular orbital, LUMO) orbitals of the Pc core. At higher concentrations, a subtle shoulder or minor peak preceding the Q band can be discerned, which is attributed to the formation of Pc dimers [42]. However, the monomeric form of these compounds exhibits significantly stronger absorption, suggesting that the bulky peripheral substituents—such as the 4i (5-isopropyl-2-methylphenoxy) group—effectively hinder π–π stacking and molecular aggregation in solution through steric hindrance.

The broader B band, on the other hand, results from π–π* transitions originating from deeper-lying molecular orbitals to the LUMO. The position and intensity of this band are also influenced by the central metal atom, which can modulate the electron distribution within the macrocyclic framework. Notably, the central metal ion plays a pivotal role in determining the precise location of the Q band maximum, which undergoes a blue shift (i.e., shifts toward shorter wavelengths), following the order NiPc < CoPc < CuPc < ZnPc. This trend can be rationalized by considering the varying electron-withdrawing effects of the central metal ions. More polarizing metals more effectively stabilize the e_g_ orbital, thereby increasing the energy gap between the HOMO and LUMO. This, in turn, shifts the absorption to higher energies, corresponding to shorter wavelengths.

### 3.3. Raman Spectra of MPcs with Different Substituents

Pcs can undergo substitution reactions at their peripheral positions, where it can be functionalized with either electron-withdrawing or electron-donating groups. This versatility leads to the formation of a wide array of Pc derivatives, each exhibiting distinct physical, chemical, thermal, optical, and electrical properties, as documented in references [22,50,51,52,53,54]. To investigate the impact of various substituents on the Raman shifts in Pc molecules, we collected Raman spectra of Pc compounds modified with different types of substituents: electron-donating groups (e.g., –-NH_2_), electron-withdrawing groups (e.g., –NO_2_), and bulky substituents (e.g., C_10_H_13_O–). Through this systematic approach, we can analyze in detail how the electronic effects and steric hindrance effects of these substituents influence the molecular vibration modes of Pc.

**Figure 3:**
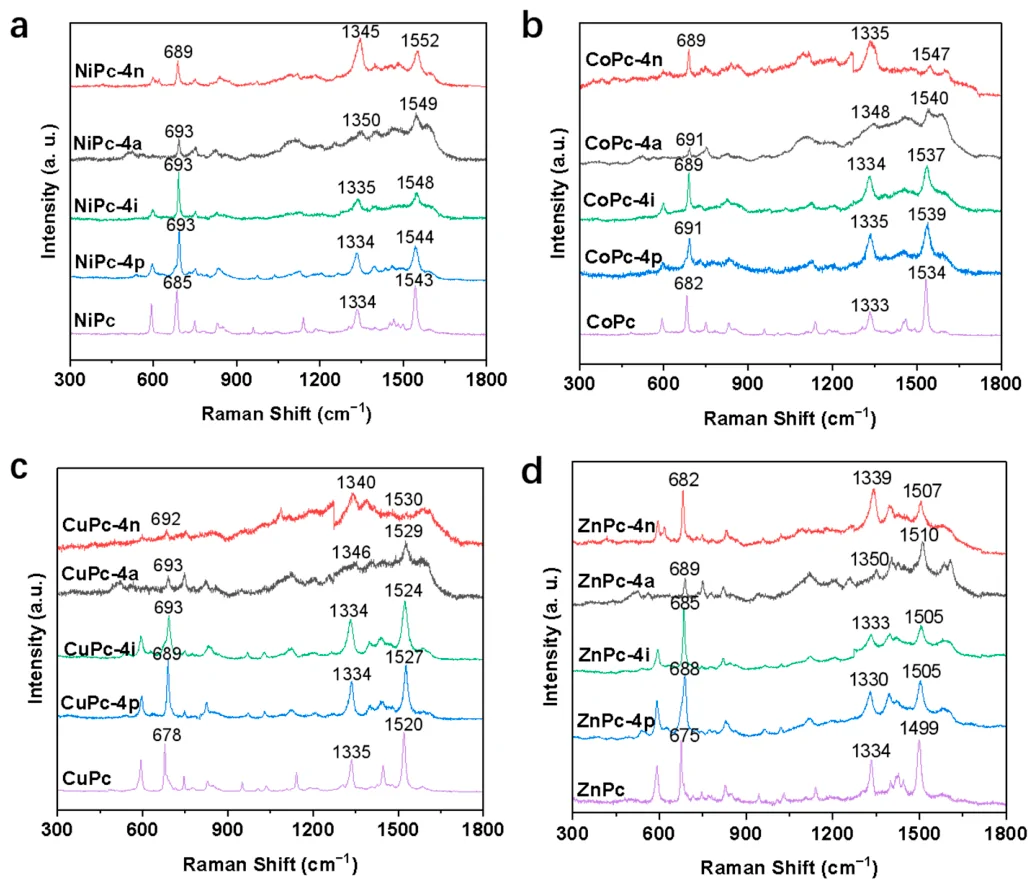
Influence of peripheral substituents on the Raman spectra of MPcs with amino (–NH_2_), nitro (–NO_2_), p-tert-butylphenoxy, and 5-isopropyl-2-methylphenoxy groups. (**a**) NiPc derivatives. (**b**) CoPc derivatives. (**c**) CuPc derivatives. (**d**) ZnPc derivatives.

As illustrated in Figure 3, it is evident that different substituents exert distinct effects on the Raman peaks of Pc compounds. The peaks at ~690 cm^−1^, ~1330 cm^−1^, and ~1530 cm^−1^ corresponding to characteristic marker modes of the phthalocyanine macrocycle were detailed comparison. Notably, the bands at ~690 cm^−1^ exhibit minimal variation across different substituents of MPcs, but all have greater Raman Shifts than that MPc with unsubstituents, suggesting that the substituents have a relatively influence on the macrocycle breathing. The same as the band at ~1530 cm^−1^, while the band at ~1530 cm^−1^ reveals a clear trend, for each metal center except Zn, the Raman shifts follow the order substituents with increasing electron-withdrawing ability (e.g., –NO_2_ > –NH_2_ > bulky groups), i.e., NiPc-4n > NiPc-4a > NiPc-4i > NiPc-4p, CoPc-4n > CoPc-4a > CoPc-4p > CoPc-4i, CuPc-4n > CuPc-4a > CuPc-4p > CuPc-4i, and ZnPc-4a > ZnPc-4n > ZnPc-4i = ZnPc-4p. These observations indicate that substituents affect the vibrations of the Pc ring through inductive and conjugative effects, modifying the electron density and enhancing stability.

Furthermore, electron-donating groups such as –NH_2_ increase the electron cloud density, weakening the vibrational characteristics of the chemical bond, while electron-withdrawing groups like –NO_2_ reduce the electron cloud density and strengthen the vibrational characteristics. Consequently, the bands for NiPc, CoPc, and CuPc with –NO_2_ substituents exhibit higher Raman shifts compared to those with –NH_2_. However, for ZnPc, the trend differs because Zn^2+^ lacks the ability to form effective intramolecular conjugation with the Pc macrocycle, as its d orbitals are fully occupied and do not participate in the π-conjugation system. Interestingly, the substituents have the most significant impact on the ~1330 cm^−1^ band, corresponding to in-plane bending of C–N. The maximum and minimum shifts for the same central metal can differ by up to 20 cm^−1^, showing higher sensitivity to substituent effects than to the central metal. Among them, the Raman shifts in MPc derivatives with –NO_2_ and –NH_2_ substituents are higher than those with C_10_H_13_O–or no substituents. The influence of substituents on Raman shifts can be understood in terms of two competing effects: (1) Electronic effects (inductive + resonance): electron-withdrawing groups (–NO_2_) decrease electron density on the Pc ring, increasing bond vibrational characteristics and causing blue shifts. Electron-donating groups (–NH_2_) enhance electron density but may also strengthen conjugation, leading to comparable or slightly lower shifts. (2) Steric effects: bulky substituents such as C_10_H_13_O– groups tend to adopt non-planar conformations relative to the Pc ring, reducing π-conjugation efficiency. This weakens electronic coupling and results in smaller Raman shifts.

Thus, the observed trend reflects a balance between electronic conjugation strength and steric hindrance. Electron-withdrawing groups (e.g., –NO_2_) reduce electron density and increase bond stiffness, resulting in blue shifts, whereas bulky substituents weaken conjugation and lead to relatively lower frequencies. These findings provide valuable insights into the structure-property relationships of substituted Pc compounds and their potential applications in various fields.

### 3.4. Influence of Substituent Positions on Raman Spectra

**Figure 4:**
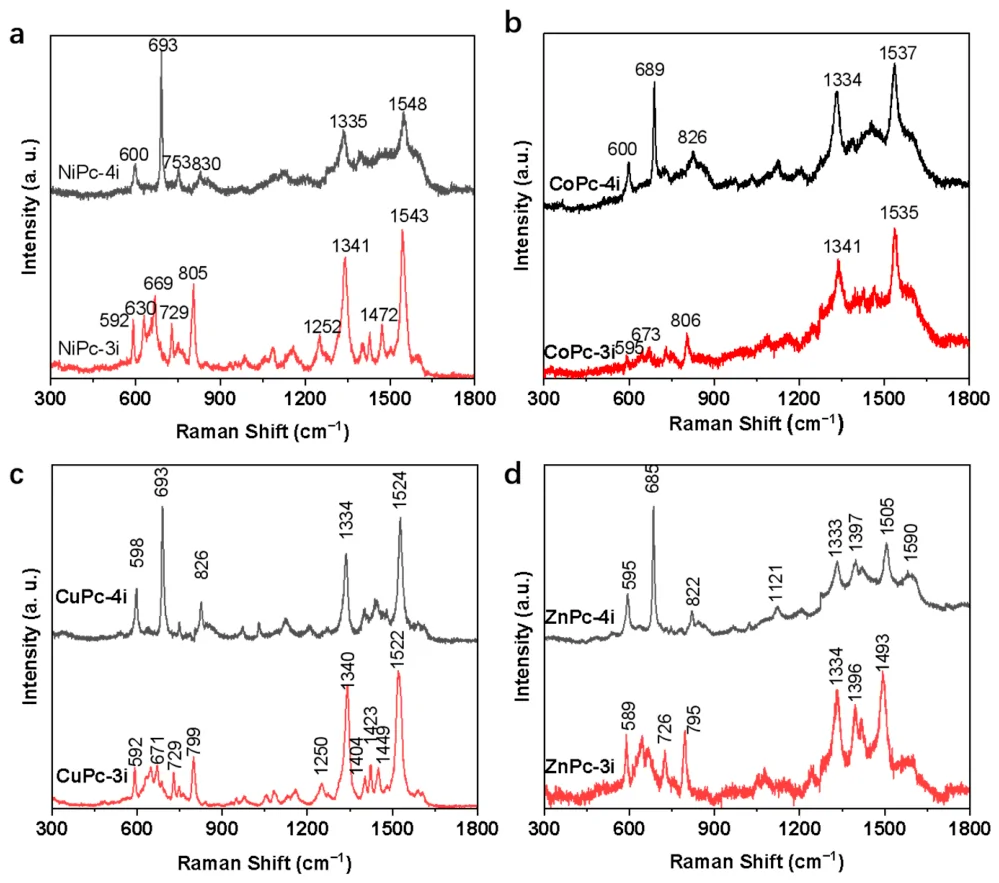
Comparative Raman spectral analysis of MPcs with 5-isopropyl-2-methylphenoxy substituents at 3- and 4-positions. (**a**) NiPc derivatives. (**b**) CoPc derivatives. (**c**) CuPc derivatives. (**d**) ZnPc derivatives.

The substitution position on the Pc ring also plays a significant role in modulating Raman spectral features. To examine this effect, MPcs bearing 5-isopropyl-2-methylphenoxy groups at three and four positions were synthesized and analyzed (Figure 4). Distinct differences are observed in the Raman spectra depending on the substitution position. In the region ~690 cm^−1^, the four-substituted compounds exhibit a single, sharp peak, such as 693 cm^−1^ for NiPc-4i, while the three-substituted counterparts display a broader distribution or cluster of peaks, for example, 630 cm^−1^, 669 cm^−1^, and 729 cm^−1^ for NiPc-3i. Similarly, in the lower wavenumber region near 600 cm^−1^, the Raman shifts of four-substituted MPcs are consistently higher than those of the three-substituted analogs. For instance, 600 cm^−1^ (NiPc-4i) > 592 cm^−1^ (NiPc-3i). A comparable trend is observed near 1500 cm^−1^, where four-substitution generally results in a higher Raman shift than three-substitution. Interestingly, near 1330 cm^−1^, the trend reverses slightly, the three-substituted compounds exhibit marginally higher Raman shifts than the four-substituted ones. These variations can be attributed to differences in how the substitution position affects molecular symmetry and electronic coupling. Three-position substitution typically occurs at the meta-position of the benzene ring relative to the Pc core. This substitution tends to cause less disruption to molecular symmetry and results in more localized perturbations. In contrast, four-position substitution occurs at the para-position, which allows for stronger coupling with the π-electronic system of the Pc core. This stronger interaction leads to more extensive electron cloud redistribution and more significant changes in the molecular vibrational profile.

From an electronic perspective, substituents at the four-position are more likely to resonate with the delocalized π-system of the Pc macrocycle. This resonance increases the extent of electronic delocalization and can substantially modify the molecular polarizability, which is the fundamental basis of Raman scattering. On the other hand, three-position substituents are spatially and electronically more removed from the core conjugation pathway, leading to weaker coupling and more localized effects. Thus, substitution at the four-position generally exerts a greater influence on both the symmetry and polarizability of the molecule, resulting in stronger Raman activity and shifted peak positions, whereas three-position substitution causes more subtle and localized spectral changes. 

Overall, the Raman spectral features of MPc are highly sensitive to changes in the molecular structure, particularly the nature of the central metal ion and the type and position of substituents. These spectral signatures provide valuable structural and electronic information, which is crucial for understanding the physicochemical behavior of MPc and optimizing their performance in various applications such as sensing, catalysis, and optoelectronics.

## 4. Conclusions

In conclusion, the vibrational properties of MPcs are governed by a complex interplay of central metal effects, substituent electronics, and substitution topology. Central metals modulate Raman shifts primarily through changes in metal-ligand bond strength, electronic configuration, and polarizability, resulting in a systematic shift sequence (Ni > Co > Cu > Zn). Peripheral substituents influence vibrational behavior through inductive and conjugative effects, while steric factors may contribute to variations in the electronic distribution within the phthalocyanine macrocycle. Importantly, the substitution position significantly affects the observed resonance Raman response, with four-substituted derivatives exhibiting stronger interactions with the conjugated system and more pronounced spectral changes than their three-substituted counterparts. Overall, this work establishes a clear and correlation between molecular structure, electronic distribution, and Raman response, providing valuable insights for the structural characterization and rational design of phthalocyanine-based micro/nanostructured and interfacial functional materials.

## Figures and Tables

**Table 1:** Different N–M bond lengths for MPcs with various central metal.

	NiPc (Å)	CoPc (Å)	CuPc (Å)	ZnPc (Å)
N–M-4i	1.826	1.836	1.846	1.926
N–M [29]	1.90	1.92	1.95	1.99
N–M [45]	~1.88–1.90	1.92	1.95	1.98
N–M [28]	1.91		1.96	2.00
N–M [46]	1.91			

## Data Availability

All data needed to support the conclusions in the paper are presented in the manuscript. Additional data related to this paper may be requested from the corresponding author upon request.
